# Regulation of adiposity by mTORC1

**DOI:** 10.1590/S1679-45082017RB4106

**Published:** 2017

**Authors:** Juliana Magdalon, William Tadeu Festuccia

**Affiliations:** 1 Hospital Israelita Albert Einstein, São Paulo, SP, Brazil; 2 Universidade de São Paulo, São Paulo, SP, Brazil

**Keywords:** Obesity, Adiposity, Adipocytes, Lipids, Thermogenesis, Obesidade, Adiposidade, Adipócitos, Lipídeos, Termogênese

## Abstract

Obesity is characterized by an excessive increase in the adipose tissue mass, and is associated with higher incidence of several chronic metabolic diseases, such as type 2 diabetes. Therefore, its increasing prevalence is a public health concern, and it is important to better understand its etiology to develop new therapeutic strategies. Evidence accumulated over the years indicates that obesity is associated with a marked activation in adipose tissue of the mechanistic target of rapamycin complex 1 (mTORC1), a signaling pathway that controls lipid metabolism, and adipocyte formation and maintenance. Curiously, mTORC1 is also involved in the control of nonshivering thermogenesis and recruitment as well as browning of white adipose tissue. In this review, we explored mTORC1 functions in adipocytes and presented evidence, suggesting that mTORC1 may either increase or reduce adiposity, depending on the conditions and activation levels.

## RESUMO

A obesidade é caracterizada pelo aumento excessivo da massa de tecido adiposo, estando associada à maior incidência de diversas doenças metabólicas crônicas, como diabetes tipo 2. Sua crescente prevalência é uma questão de saúde pública, e faz-se importante compreender melhor sua etiologia, para desenvolver novas estratégias terapêuticas. As evidências acumuladas por muitos anos indicam que a obesidade está associada à significativa ativação no tecido adiposo do complexo 1 da proteína alvo mecanístico da rapamicina (mTORC1), uma via de sinalização que regula o metabolismo de lipídeos, bem como a formação e manutenção de adipócitos. Curiosamente, mTORC1 também está envolvido no controle da termogênese, independente do tremor muscular, e no recrutamento e *browning* de tecido adiposo branco. Nesta revisão, exploramos as diferentes funções do mTORC1 em adipócitos e apresentamos evidências que sugerem que o mTORC1 pode aumentar ou reduzir a adiposidade, dependendo das condições e de seu nível de ativação.

## INTRODUCTION

Obesity is the excessive accumulation of fat in adipose tissue and ectopically in other organs, such as in the liver, resulting from chronic periods of positive energy balance, characterized by energy intake higher than energy expenditure. Clinically, patients are considered obese after reaching a body mass index (BMI) equal or higher than 30kg/m^2^. The prevalence of obesity has dramatically increased in the last decades attaining levels of a global pandemic,^(^ ^1^ ^)^ such a worrisome event that has been mainly attributed to the current lifestyle characterized by unhealthy dietary habits and sedentary behavior. A major concern for public health is the fact that obesity is frequently associated with the development of several chronic diseases, such as type 2 diabetes,^(^ ^2^ ^)^ cardiovascular diseases,^(^ ^3^ ^)^ hepatic steatosis^(^ ^4^ ^)^ and some types of cancer.^(^ ^5^ ^)^ Importantly, despite being an important risk factor to metabolic disorders, not all obese people develop those diseases. Indeed, obese patients that accumulate fat preferentially in the visceral adipose depots located inside the peritoneal cavity have a much higher chance of developing metabolic disorders than those that accumulate fat in subcutaneous adipose depots.^(^ ^6^ ^)^ Such a phenomenon has been related to the higher spillover of lipids, lipotoxicity and inflammatory process associated with visceral, but not subcutaneous obesity.

### Types of adipocytes and factors driving adipose tissue enlargement

**Figure 1:**
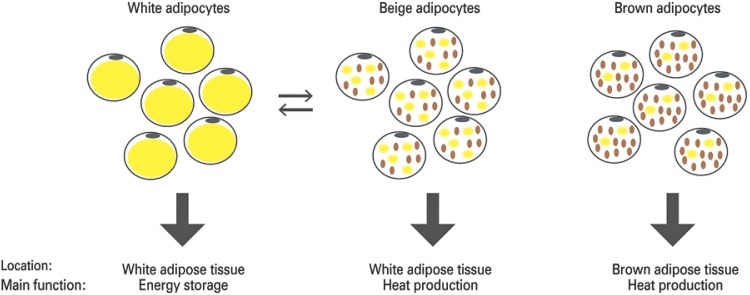
Differences among white, beige and brown adipocytes in relation to location and main function Yellow spots: lipid droplets; brown spots: mitochondria; black spots: nuclei.

Three different types of adipocytes can be found in humans: white adipocytes, the main cells composing white adipose tissue (WAT), that exert lipid storage and endocrine functions; brown adipocytes, the main cells composing brown adipose tissue (BAT), whose main function is to produce heat through mitochondrial uncoupling protein 1 (UCP-1)-dependent nonshivering thermogenesis; and beige adipocytes, which are located in white adipose depots and, depending on the condition, may act either as white (lipid storage) or brown (UCP-1-induced heat production) adipocytes (Figure 1).^(^ ^7^ ^)^

Excessive fat accumulation and adipose tissue enlargement generally result from a combination of increase in both white adipocyte diameter (hypertrophy) and number (hyperplasia). While the former is determined by cell content of triacylglycerol (TAG), which reflects the balance between lipolysis (triacylglycerol hydrolysis) and lipogenesis (triacylglycerol synthesis), the latter reflects the equilibrium between cell formation, which involves the proliferation, commitment and differentiation of adipose tissue-resident mesenchymal cells into mature adipocytes, and adipocyte apoptosis.

Over the years, some studies have shown strong evidence that the mechanistic target of rapamycin complex 1 (mTORC1) is an important regulator of adipose tissue formation and lipid storage function. Indeed, mTORC1 has been implicated in the regulation of early adipocyte precursor commitment, preadipocyte differentiation into mature adipocytes (adipogenesis), as well as TAG synthesis and mobilization in adipocytes.^(^ ^8^ ^)^ Moreover, the activity of mTORC1 was consistently shown to be increased in adipose tissue of genetically and high fat diet-induced obese mice,^(^ ^9^ ^)^ indicating a likely involvement of this complex in adipose tissue enlargement. In addition to its role in white fat, recent studies have also shown that mTORC1 is involved in the regulation of BAT thermogenesis and beige cells recruitment and activation (browning). Herein, we reviewed the major findings of those studies and propose a model of adiposity regulation by mTORC1.

### mTORC1 biology

**Figure 2:**
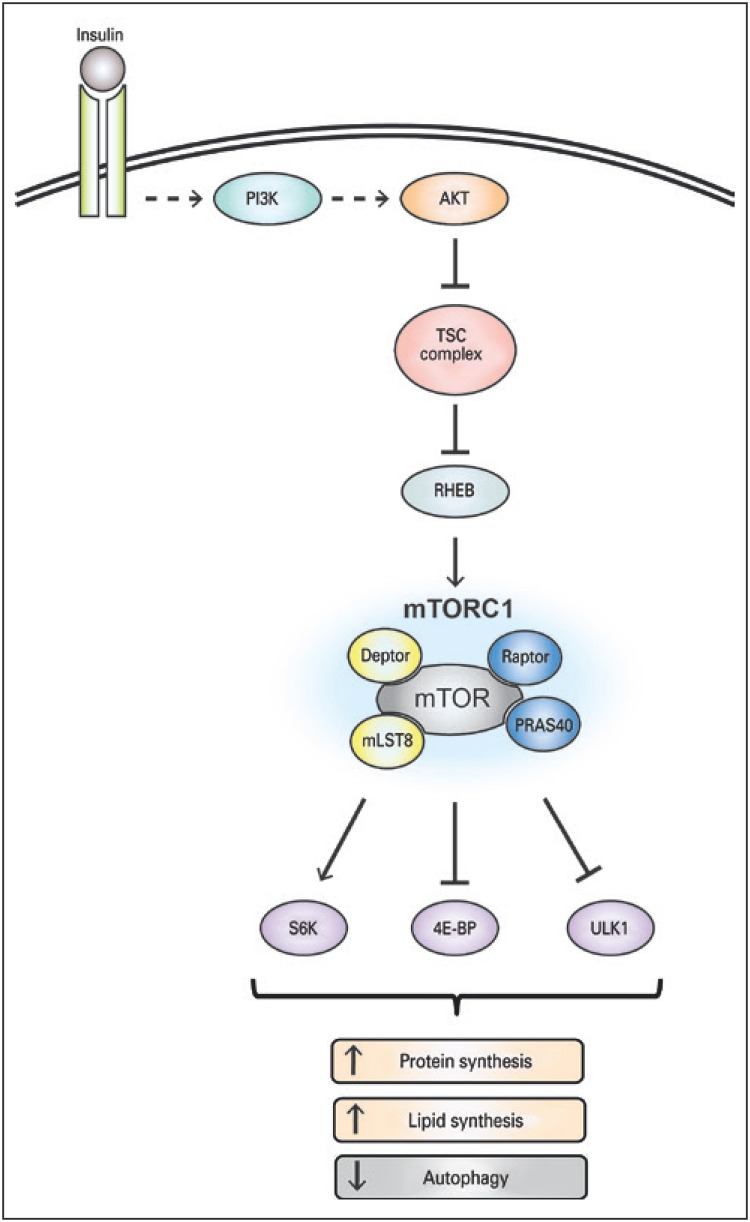
Overview of mechanistic target of rapamycin complex 1 signaling pathway and function (mTOR)

mTORC1 is composed of several proteins in addition to the serine/threonine kinase mTOR: regulatory-associated protein of mTOR (Raptor), mammalian lethal with SEC13 protein 8 (mLST8), proline-rich AKT substrate of 40kDa (PRAS40) and DEP domain-containing mTOR-interacting protein (DEPTOR). mTORC1 plays a key role in the regulation of cell metabolism and homeostasis. When activated, mTORC1 promotes anabolic processes, such as protein, nucleotide and lipid syntheses, and inhibits catabolic processes, such as autophagy, through the phosphorylation of several proteins, including S6 kinases (S6Ks), eukaryotic initiation factor 4E (eIF4E)-binding proteins (4E-BPs) and unc-51 like autophagy activating kinase 1 (ULK-1), among others. mTORC1 is mainly activated by amino acids through a process mediated by proteins denominated Rags, which involves complex translocation from the cytosol to the lysosomes.^(^ ^10^ ^)^ In addition to amino acids, mTORC1 is also activated by growth factors such as insulin, through the canonical IRS-PI3K-AKT signaling pathway. Upon its activation, AKT phosphorylates and inhibits the tuberous sclerosis complex (TSC), which is a GTPase-activating protein (GAP) toward Ras homolog enriched in brain (RHEB). When TSC is inhibited and, consequently, RHEB is bound to GTP, it stimulates mTORC1 recruitment to the lysosome membrane, where it is activated in the presence of amino acids (Figure 2).^(^ ^11^ ^)^

### mTORC1 loss-of-function and adiposity

The first evidence indicating that mTORC1 plays an important role in the regulation of adiposity came out from *in vitro* studies showing that pharmacological inhibition of mTORC1 with rapamycin completely blocks the ability of 3T3-L1 cells to differentiate into mature adipocytes. These effects were attributed to a reduced expression of peroxisome proliferator-activated receptor (PPAR)γ and CCAAT-enhancer-binding protein (C/EBP)α, which are major transcription factors required for adipogenesis.^(^ ^12^ ^–^ ^15^ ^)^ Besides these *in vitro* findings, the administration of rapamycin to rodents *in vivo* was associated with a reduction in adiposity due to lower expression of PPARγ and target genes involved in lipid uptake and storage^(^ ^16^ ^)^ and a protection against the fat mass expansion and obesity induced by the intake of a high-fat diet.^(^ ^17^ ^)^

Genetically modified mice displaying whole-body or tissue specific (Cre-lox) deficiencies of mTORC1 essential component *Raptor,* or of the downstream substrate *S6k* were also employed to investigate mTORC1 involvement in the regulation of adiposity. Corroborating studies with rapamycin, mice with whole-body deficiency of *S6k1* displayed reduced fat mass, and were protected against diet-induced obesity;^(^ ^9^ ^)^ such phenotypes were attributed to impaired adipogenesis.^(^ ^18^ ^)^ Nonetheless, a similar reduction in adiposity and protection against obesity was also seen in mice with *Raptor* deletion in adipocytes.^(^ ^19^ ^,^ ^20^ ^)^ Conflicting molecular mechanisms were provided to explain those phenotypes, *i.e*., either enhanced energy expenditure as a result of adipocyte browning,^(^ ^19^ ^)^ or a defect in adipose tissue expansion due to impaired C/EBPα expression.^(^ ^20^ ^)^ The above differences, however, likely reflect the use of distinct *Fabp4* (*ap2*) *and,* adiponectin promoters to drive Cre recombinase expression, with the former showing a much lower adipocyte specificity than the latter.^(^ ^21^ ^)^ Finally, mice with deficiency of both mTORC1 and 2 in adipocytes due to the adiponectin-cre drive deletion of *mTOR* have also reduced adiposity, which was associated with adipocyte browning and impaired adipogenesis as a result of reduced PPARγ and C/EBPα expression.^(^ ^22^ ^)^ Therefore, it is possible to conclude that mTORC1 deficiency or complete inhibition in adipocytes leads to impaired adipogenesis and lipid deposition, enhanced adipocyte browning and reduced adiposity.

### mTORC1 partial inhibition and adiposity

In sharp contrast to the impaired adipogenesis and reduction in adiposity induced by adipocyte mTORC1 deficiency or pharmacological inhibition, partial inhibition of this complex was shown to enhanced adipogenesis and increase adiposity. Indeed, partial knockdown of mTOR with a shRNA potentiates the differentiation of 3T3-L1 preadipocytes into mature adipocytes *in vitro,* as evidenced by the higher accumulation of TAG.^(^ ^23^ ^)^ Furthermore, partial inhibition of mTORC1 activity *in vitro* and *in vivo* through the overexpression of *Deptor,* an endogenous inhibitor of mTORC1, enhanced adipogenesis and exacerbated increase in body weight and WAT mass induced by the intake of a high-fat diet.^(^ ^24^ ^)^ On the mechanistic level, such enhanced adipogenesis and increased adiposity induced by partial mTORC1 inhibition were attributed to the dampening of the negative feedback upon IRS function and intracellular insulin signaling exerted by mTORC1/S6K1, which then enhanced the PI3K-AKT-PPARγ pathway and, consequently, the adipogenesis and lipogenesis.^(^ ^24^ ^)^ Therefore, partial mTORC1 inhibition promotes adipogenesis and adipose tissue expansion.

### mTORC1 gain-of-function and adiposity

Our group was the first to characterize the effect of adipocyte constitutive mTORC1 activation on adiposity and body weight *in vivo.* Indeed, mice with constitutive mTORC1 activation in adipocytes displayed a depot-specific reduction in the mass of visceral adipose tissue (retroperitoneal depot), in association with increased browning (UCP-1 content), lipolysis, mitochondrial mass and oxidative activity.^(^ ^25^ ^)^ In contrast to those findings, however, constitutive mTORC1 activation in adipocytes using the nonadipocyte-specific *Fabp4* Cre mice reported no alteration in WAT mass in 2-day-old mice.^(^ ^26^ ^)^ It is worth mentioning that older mice were not evaluated in this study since the animals died within 48 hours after birth, probably due to *Tsc1* deletion in cells other than adipocytes, mediated by non-specific Cre expression.^(^ ^26^ ^)^ Based on those findings, one may conclude that constitutive mTORC1 activation in adipocytes reduces visceral adiposity.

### Proposed model of adiposity regulation by mTORC1

**Figure 3:**
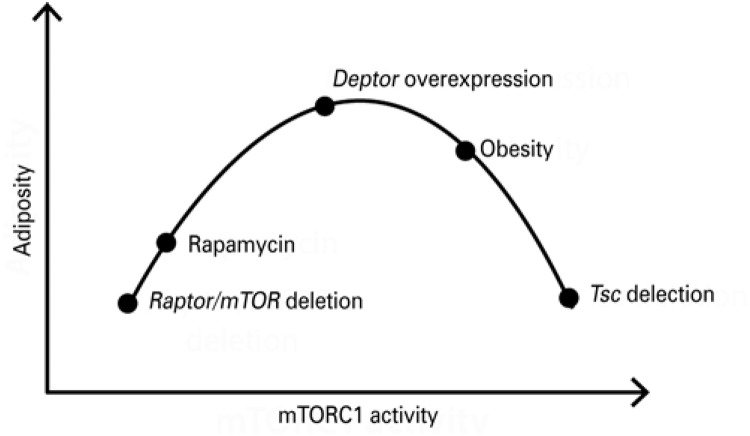
Association between mechanistic target of rapamycin complex 1 (mTORC1) activity and adiposity in different animal models

Based on the above-detailed studies describing the changes in adiposity associated with mTORC1 loss-of-function, partial inhibition, and constitutive activation, we and other authors^(^ ^27^ ^)^ have proposed that mTORC1 regulation of adiposity follows an inverted U-shaped curve, in which optimal levels of mTORC1 activity - neither too low as induced by *Raptor* deletion and rapamycin treatment, not too high as that induced by *Tsc1* deletion – are required for this complex to promote its pro-adipogenic and lipogenic actions (Figure 3). Such optimal level of mTORC1 activity could respond for the lipid accretion and exacerbated adiposity found upon obesity and *Deptor* overexpression, for example. Studies evaluating the effects of different levels of mTORC1 activity upon adiposity in a unique experiment setting, however, are still required to confirm this proposed model.

### mTORC1 and nonshivering thermogenesis

In addition to its role in the regulation of adiposity, mTORC1 was also shown to be necessary for BAT thermogenic function and WAT browning induced by β3-adrenergic stimulation or cold exposure.^(^ ^28^ ^–^ ^30^ ^)^ More specifically, mTORC1 is activated in BAT and WAT in response to cold, and β-adrenergic stimulation through a mechanism that seems to involve a β-adrenergic receptor-mediated increase in cAMP and activation of protein kinase A (PKA), which in turn phosphorylates Raptor and mTOR, increasing complex activity. Moreover, in both BAT and visceral WAT, mTORC1 was shown to regulate mitochondrial biogenesis, oxidative metabolism and UCP-1 expression.^(^ ^25^ ^,^ ^28^ ^)^ Altogether, these findings establish mTORC1 as an important regulator of nonshivering thermogenesis in both BAT and beige cells.

## CONCLUSION

Obesity is a current concern worldwide due to its increasing prevalence and strong association with several metabolic diseases. Unfortunately, up to date, no effective treatment has been established to counteract the development of these diseases. mTORC1 signaling is activated in adipose tissue upon obesity and participates in the enhanced lipid deposition and adipose tissue expansion found in this condition. Understanding the mechanisms by which different levels of mTORC1 activity regulate adiposity may enable the development of strategies to efficiently counteract adipose tissue enlargement and obesity.
